# Editorial: The function of Schwann cells in peripheral nervous system

**DOI:** 10.3389/fncel.2022.1129560

**Published:** 2023-01-09

**Authors:** Bo He, Jifan Hu, Zhaowei Zhu

**Affiliations:** ^1^Joint and Orthopaedics Trauma, Department of Orthopedics, The Third Affiliated Hospital of Sun Yat-sen University, Guangzhou, China; ^2^VA Palo Alto Health Care System, Stanford University Medical School, Palo Alto, CA, United States; ^3^Key Laboratory of Organ Regeneration and Transplantation of Ministry of Education, Cancer Center, The First Hospital of Jilin University, Changchun, Jilin, China; ^4^Department of Plastic Surgery, The First Affiliated Hospital of Sun Yat-sen University, Guangzhou, China

**Keywords:** Schwann cells, Schwann-like cells, peripheral nerve, nerve regeneration, nerve injury, stem cells, tissue engineering

It is generally believed that the decline in the capacity of peripheral nerve regeneration relies on the slow regeneration of neuronal axons. However, Painter et al. believed that the key factor that affects mammalian peripheral nerve regeneration was not the neuron itself. Instead, Schwann Cells (SCs) played an important role in the process of nerve regeneration after peripheral nerve injury. First of all, SCs are necessary for successful nerve regeneration. After injury, they become partially de-differentiated, with increased expression of nerve regeneration genes and secretion of neurotrophic factors, including GDNF, NGF, BDNF, and CNTF. The proximal ends of peripheral nerves formed growth cones facilitating regeneration of nerve fiber. Secondly, SCs guide the orderly extension of nerve axons by organizing suitable scaffolds. Thirdly, SCs clear the myelin sheath fragments produced by Wallerian degeneration of injured nerves, thus removing obstacles on the regeneration channels. In order to solve shortage of primary SCs, researchers also explored the substitutes of SCs by inducing stem cells into Schwann-like cells (SLCs). Both SCs and SLCs are indispensable and ideal seed cells for tissue engineering.

Our Research Topic aimed to investigate the regulatory roles of Schwann Cells and Schwann-like cells in peripheral nervous systems. We are very grateful to the 145 contributors from 15 different countries participating in current Research Topic with six abstracts, four review articles and six original research papers. These publications discussed from different angles of the current challenges to the field. It could be summarized into the following three aspects: (1) Important genes or proteins of SCs during peripheral nerves regeneration; (2) Scaffolds or stem cells exploration in peripheral nerves; (3) SCs related Reviews.

After peripheral nerve injury, SCs, vascular endothelial cells and blood derived macrophages reacted and conducted Wallerian deformation process. He et al. performed transcriptome sequencing and qPCR verification on the sciatic nerves of rats 4 and 7 days after the clamp injury. They found that Notch1 might act as a coordinator among the processes of vascular endothelial cell regeneration, macrophage recruitment and nerve regeneration.

Shen et al. transfected CD146 siRNA into primary rat SCs, and found that cell proliferation rate decreased significantly with increased migration. These results implied that CD146, as an important cell adhesion molecule, might have a significant impact on regulating biological behavior of SCs.

Ulrichsen et al. found that Sortilin could affect Remak bundle formation. When they cultured Sort1 -/- Schwann cells *in vitro*, they found SCs lost the ability to migrate and myelination.

Chen et al. combined mesenchymal stem cell transplantation and electric acupuncture to treat acute sciatic nerve contusion in rats. After assessment of MRI, they found that the neural function recovery speed of the experimental group was much faster than that of control group.

Zhong et al. collected numerous transaction figures of peripheral nerves and established mathematical models to reproduce the contour of functional bundles inside nerves. What's more, they attempted to predict the spatial continuity of axons to the distal. Fang and Zou found that Col6, as component of extracellular matrix in nerves, could promote the formation of axon bundles. Considering safer clinical application, Col6α2 seemed able to promote orderly axon formation through NCAM1 guided pathway with lower immunogenicity than Col6 complex *in vivo*.

Manganas et al. reviewed relevant literature about how SCs adapted to mechanical properties of their external micro-environment. Rao et al. compared the survival of SCs and/or SLCs when repairing peripheral nerve injury, and their performance changes when encountering different biomaterials used in tissue engineering nerve grafts. Wei et al. summarized outcomes of transplantation of SCs and SLCs in different peripheral neuropathies, and importance of SCs in the process of repairing nerves. Su et al. discussed construction of tissue engineering nerve grafts with SCs. They compared not only the advantages of nerve scaffolds loaded with SCs, but also the related problems of SCLs induced by stem cells. In conclusion, all these publications largely enriched theoretical knowledge of importance of SCs and nerve regeneration, and proposed a new treatment strategy for better application of SCs and SLCs to peripheral nerve injury repair in the future.

In recent years, many studies have been focused on understanding the process of PNS repair and reconstruction after injury and aim to find novel clinical therapeutical methods. The advancement of genomics, epigenomics and proteomics research methods has made it possible to study the regulation of transcription, post-transcriptional modification and translation in SCs and SLCs derived from stem cells, as well as the biological roles of these cells in PNS regulation, but many cellular and molecular mechanisms are still unclear and need to be farther investigated. We hope these precious achievements could bring greater breakthroughs in the future.

We, as the guest editors group, appreciate all authors once more for their valuable contributions. It is our honor that all readers of this Research Topic will enjoy these expert opinions and excellent research results.

## Author contributions

BH, JH, and ZZ have connected them all within the editorial. All authors have contributed to this editorial and writing comments to the different articles.

